# Risk factors of embolism for the cardiac myxoma patients: a systematic review and metanalysis

**DOI:** 10.1186/s12872-020-01631-w

**Published:** 2020-07-25

**Authors:** Yanna Liu, Jiwei Wang, Liangyun Guo, Luyi Ping

**Affiliations:** Department of Ultrasound, The Second Affiliated Hospital of Nanchang University, Minde Road No.1, Nanchang, 330006 Jiangxi China

**Keywords:** Cardiac myxoma, Embolism, Risk factors, Meta-analysis

## Abstract

**Background:**

The risk factors contributing to embolism in cardiac myxoma (CM) are yet controversial. This systematic review and meta-analysis aimed to clarify the risk factors of embolism for the CM patients.

**Methods:**

PubMed, Embase, Cochrane library, Web of Science, China National Knowledge Infrastructure, Wan Fang, and Wei Pu databases were searched from inception to June 2019. Statistical analysis was conducted using Stata version 14.0. The pooled odds ratio or mean difference with 95% confidence interval was estimated for each risk factor.

**Results:**

Herein, 12 studies, encompassing 1814 patients, were included. The pooled results suggested that New York Heart Association (NYHA) class I/II (*P* < 0.01), hypertension (*P* = 0.03), irregular tumor surface (*P* < 0.01), tumor in atypical location (*P* = 0.01), narrow base of tumor (*P* < 0.01), and increased fibrinogen (FIB) (*P* < 0.01) are significant risk factors of embolism in CM patients. However, sex, age, body mass index, smoking, left ventricular ejection fraction, diabetes, hyperlipidemia, atrial fibrillation, valvular heart disease, coronary heart disease, tumor size, platelet count, white blood cells, and hemoglobin were not associated with embolism (all *P* > 0.05).

**Conclusions:**

NYHA class (I/II), hypertension, irregular tumor surface, atypical tumor location, the narrow base of tumor, and increased FIB were significant risk factors of embolism in CM patients. For CM patients with these factors, early surgery might be beneficial to prevent embolism.

## Background

Cardiac myxomas (CMs) are the most prevalent type of primary cardiac tumors in adults [1] that account for approximately half of all benign lesions. The manifestations of CM patients include obstruction, preoperative embolization, and constitutional symptoms. Embolization is a major and fatal complication that occurs in 20–45% of CM patients [2]; it includes cerebral embolism and peripheral embolism. Cerebral embolism accounts for about 50% of the embolic events, mainly acute stroke symptoms. Peripheral embolism may involve extremity, visceral, and coronary arteries [3]. The right-sided CMs are associated with pulmonary embolism. These embolic events are significant causes of mortality in CM patients [4]; however, the exact factors related to the occurrence of embolism are not yet clearly identified.

Although, several risk factors of embolism are recently reported [5–16], some are controversial. For instance, a previous study [17] showed that the small size of the tumor was an independent risk factor for embolism, whereas other studies neither found any association [14] nor presented a contrary conclusion [13]. Some studies reported that the male gender was associated with an increased risk of embolism [18], while others failed to find this association [6, 7]. Therefore, we conducted this systematic review with meta-analysis to clarify the risk factors of embolism in CM patients.

## Methods

### Data sources and search strategy

The Preferred Reporting Items for Systematic Reviews and Meta-Analyses statement was followed [19]. The PubMed, Embase, Cochrane library, Web of Science, China National Knowledge Infrastructure, Wan Fang, and Wei Pu databases were used for further studies. The search strings are reported in Additional file 1. Studies published up to June 30, 2019, were eligible. No language restriction was applied. Reference lists of selected studies were checked to ensure complete coverage.

### Eligibility criteria

The studies that fulfilled the following criteria were eligible for inclusion in this meta-analysis: 1) All CM patients who underwent surgical resection and were confirmed by pathological examination; 2) Patients in each study were classified into two groups (embolic and non-embolic); 3) Embolic events were diagnosed by clinical symptoms or imaging examination. If the same population was presented in more than one publication, the study with the largest sample was included.

We excluded the studies that met at least one of the following criteria: 1) Sample size < 50; 2) Abstracts from conferences, letters to the editor, and reviews; 3) Incomplete data.

### Data extraction and quality assessment

Two authors (L.P and J.W) reviewed all retrieved articles and extracted data independently. The titles and abstracts were first screened to identify potentially eligible articles, and then, full texts were read to confirm their eligibility for inclusion in this meta-analysis. The extracted data included the following information: first author, year of publication, country, study type, sample size, age, incidence of embolism, and risk factors that include patient characteristics (sex, age, New York Heart Association (NYHA) class and atrial fibrillation (AF)), tumor characteristics (tumor surface and tumor location), and hematological parameters (white blood cell (WBC) and platelet count (PLT)). The NYHA class was divided into two groups (I/II vs. III/IV). The tumor surface was classified as irregular and regular [7]. The tumor location was classified into “typical location” (tumor arise from the interatrial septum at the border of the fossa ovalis in the left atrium) and “atypical location” (tumor arise from other sites of the left atrium or in the other cardiac chambers) [20]. The extracted data were cross-checked, and any disagreements were resolved by discussion or consultation with the third author (L.G).

The quality of the included studies was assessed using the Joanna Briggs Institute (JBI) Critical Appraisal Checklist [21] for Case Series. It contains ten items, encompassing clear criteria for inclusion, the information of participants, and the statistical methods used. Each item was determined by yes, no, unclear, and not applicable.

### Statistical analysis

Stata 14.0 was used for the statistical analysis of all data. The odds ratio/mean difference (OR/MD) and 95% confidence interval (CI) were calculated using the fixed-effect model or the random effect model. The heterogeneity among studies was quantified using the Cochran’s Q test and chi-square (I^2^) test. I^2^ = 25% was considered low, 50% was moderate, and 75% was high. When I^2^ > 50% or *P*-value < 0.05 was identified for substantial heterogeneity, we used the random effect model; otherwise, a fixed-effect model was adopted. A sensitivity analysis was conducted when substantial heterogeneity was presented. Publication bias was assessed by the Egger’s test. All tests were two-sided, and the results were considered statistically significant at *P* < 0.05.

## Results

### Search results and characteristics of included studies

**Fig. 1:**
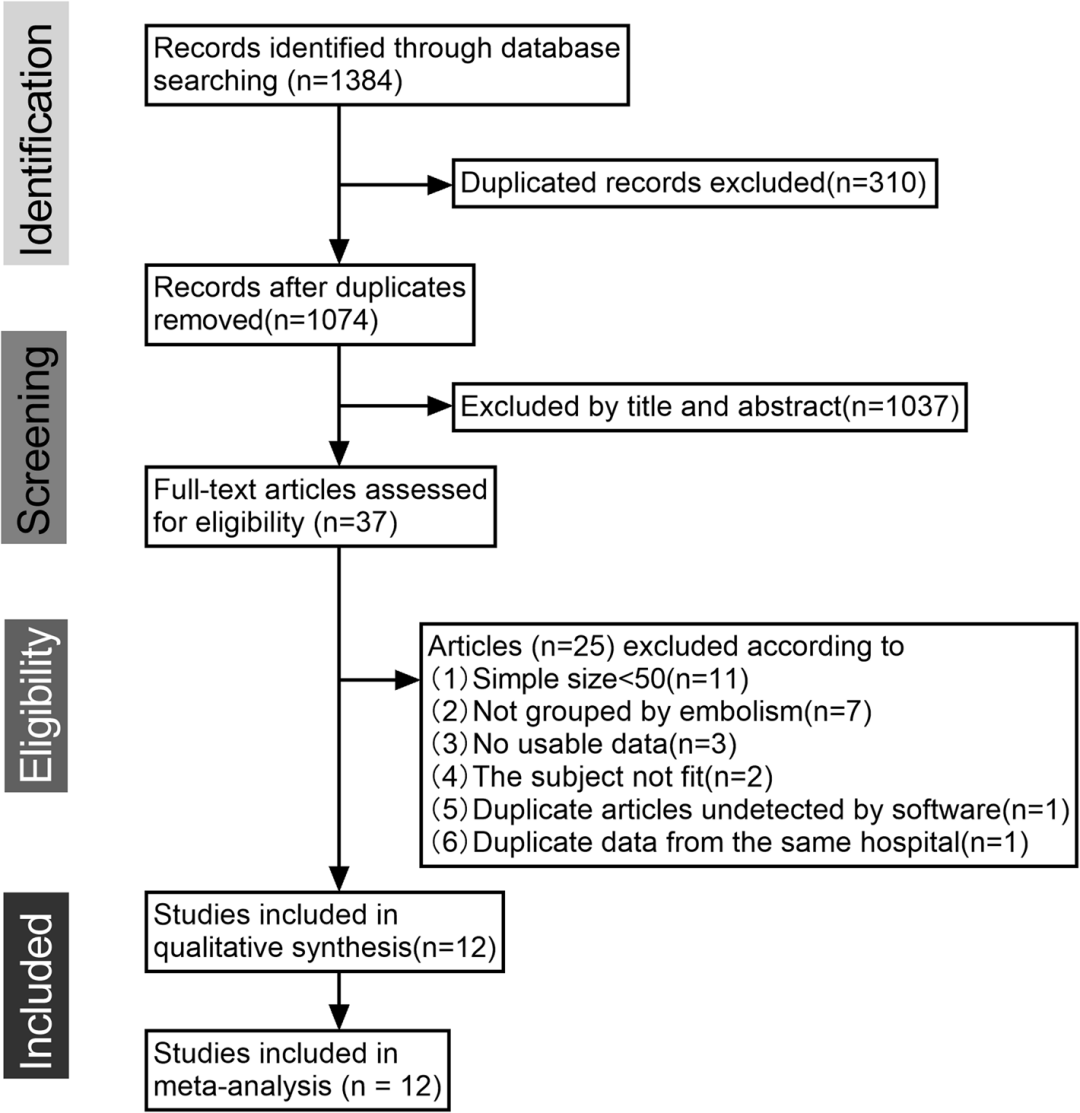
Flowchart of study selection

A total of 12 studies [5–16] encompassing 1814 patients who underwent surgery with CM were selected for the current analysis. The detailed study selection progress is shown in Fig. 1, and the main characteristics of included studies are summarized in Table 1.

**Table 1:** Characteristics of included studies

study	country	type of study	sample size		age(Mean ± SD or median(IQR))		incidence of embolism	risk factors	the JBI Appraisal Checklist
			embolisim group	non-embolism group	embolisim group	non-embolism group			
Cao 2017 [5]	China	Retrospective case series	24	87	36.0 ± 8.1	36.4 ± 6.5	21.62%	tumor size	yes(6)no(3)unclear(1) not applicable(0)
Deng 2015 [6]	China	Retrospective case series	33	129	48(38–61)	54(46–63)	20.37%	tumor location, tumor surface, MPV, PLT	yes(8)no(1)unclear(1) not applicable(0)
Lee 2012 [7]	Korea	Retrospective case series	13	46	59.2 ± 12.6	58.3 ± 12.6	22.03%	tumor surface	yes(7)no(2)unclear(1) not applicable(0)
Wang 2016 [8]	China	Retrospective case series	32	175	39.7 ± 16.6	45.0 ± 15.5	15.46%	tumor size, tumor surface	yes(7)no(2)unclear(1) not applicable(0)
Yin 2016 [9]	China	Retrospective case series	143	322	59.4 ± 10.9	52.3 ± 12.4	44.41%	age, BMI, tumor size, tumor surface, tumor location, LAD CHA2DS2–VASc score	yes(8)no(1)unclear(1) not applicable(0)
Li 2018 [10]	China	Retrospective case series	27	158	48.19 ± 13.11	49.39 ± 14.97	14.59%	tumor location,tumor base size, FIB	yes(7)no(2)unclear(1) not applicable(0)
Xu 2016 [11]	China	Retrospective case series	26	156	58.54 ± 12.65	57.29 ± 11.11	14.29%	tumor surface, tumor base size, FIB	yes(8)no(1)unclear(1) not applicable(0)
Boyacıoğlu 2017 [12]	Turkey	Retrospective case series	25	74	49 ± 16.89	50.12 ± 15.71	25.26%	AF, tumor surface, tumor size	yes(7)no(2)unclear(1) not applicable(0)
Kalçık 2019 [13]	Turkey	Retrospective case series	13	80	50(36–62)	56(45–65)	13.98%	LAD, AF, tumor size, tumor surface	yes(7)no(2)unclear(1) not applicable(0)
Ping 2019 [14]	China	Retrospective case series	32	75	54.66 ± 13.21	51.72 ± 13.76	29.91%	tumor surface, tumor location	yes(7)no(2)unclear(1) not applicable(0)
Canga 2017 [15]	Turkey	Retrospective case series	13	53	51.1 ± 11.4	55.9 ± 12.4	19.70%	sex,tumor location,tumor surface	yes(7)no(2)unclear(1) not applicable(0)
Zheng 2014 [16]	China	Retrospective case series	15	63	49 ± 9	52 ± 6	19.23%	tumor surface	yes(7)no(2)unclear(1) not applicable(0)

*SD*: standard deviation IQR: interquartile range LAD: left atrium diameter the CHA2DS2-VASc: congestive heart failure, hypertension, age ≥ 75 (doubled), diabetes, stroke(doubled), vascular disease, age 65–74, and sex category (female) recommended by European Society of Cardiology (ESC) is an easy-to-remember means of assessing stroke risk of patients with AF

### Meta-analysis of risk factors

Based on the data available from the included studies, the risk factors were classified as patient characteristics, tumor characteristics, and hematological parameters in the current review. A total of 20 risk factors were individually analyzed using a fixed-effect or a random effect model to estimate the association with embolism in CM patients. The main characteristics of each risk factor are summarized in Table 2.

**Table 2:** Main characteristics of each risk factor

Risk Factors	No. Studies	Effect Model	I^2^, %	*P*h	MD/OR	Effect Size (95% CI)	*P*
sex(male)	12	F	16.8	0.28	OR	1.21(0.96–1.53)	0.11
age	9	R	76.8	< 0.01	MD	-0.04(−3.64–3.56)	0.94
BMI	4	R	83.7	< 0.01	MD	1.21(−0.43–2.84)	0.18
NYHA class(I/II)	4	F	0.0	0.61	OR	2.98(1.66–5.33)	< 0.01^*^
smoking	6	F	0.0	0.44	OR	0.90(0.64–1.28)	0.56
LVEF	6	F	7.0	0.37	MD	0.59(−0.20–1.38)	0.14
hypertension	8	F	0.1	0.43	OR	1.41(1.04–1.92)	0.03^*^
diabetes	8	F	0.0	0.73	OR	1.32(0.89–1.94)	0.16
hyperlipidemia	6	F	0.0	0.79	OR	0.99(0.53–1.85)	0.96
atrial fibrillation	7	F	48.5	0.07	OR	1.25(0.88–1.80)	0.22
valvular heart disease	3	F	0.0	0.62	OR	0.76(0.41–1.40)	0.38
coronary heart disease	3	R	84.2	< 0.01	OR	0.99(−0.87–2.85)	0.32
tumor surface(irregular)	11	F	40.4	0.08	OR	3.99(3.04–5.25)	< 0.01^*^
tumor size	8	R	83.3	< 0.01	MD	-0.10(−0.76–0.57)	0.78
tumor location(atypical)	4	F	15.9	0.31	OR	1.81(1.13–2.88)	0.01^*^
tumor base size	2	F	0.0	0.66	MD	−0.36(−0.51--0.22)	< 0.01^*^
PLT	3	F	0.0	0.83	MD	9.95(−6.02–25.91)	0.22
WBC	4	F	34.6	0.21	MD	0.18(−0.33–0.68)	0.49
HB	4	F	0.0	0.40	MD	1.65(−2.91–6.21)	0.48
FIB	2	F	0.0	0.57	MD	0.62(0.28–0.95)	< 0.01^*^

F: fixed-effects model R: random-effects model Ph: *P* value of heterogeneity *P < 0.05

### Patient characteristics

**Fig. 2:**
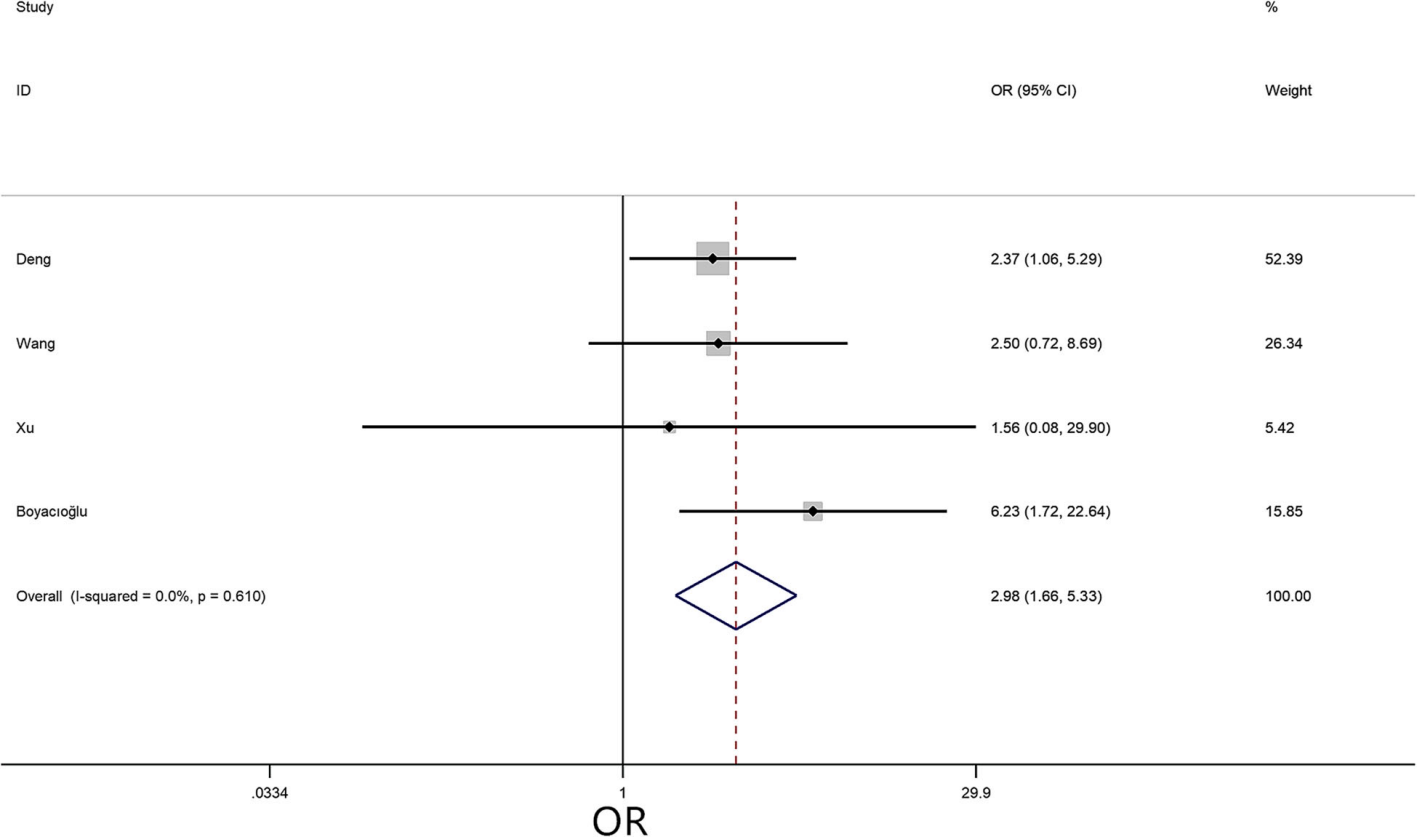
Forest plot for NYHA class between embolism: 4 studies were included, I^2^ = 0.0%, fixed-effect model was adopted; the result showed NYHA I/II is a risk factor

**Fig. 3:**
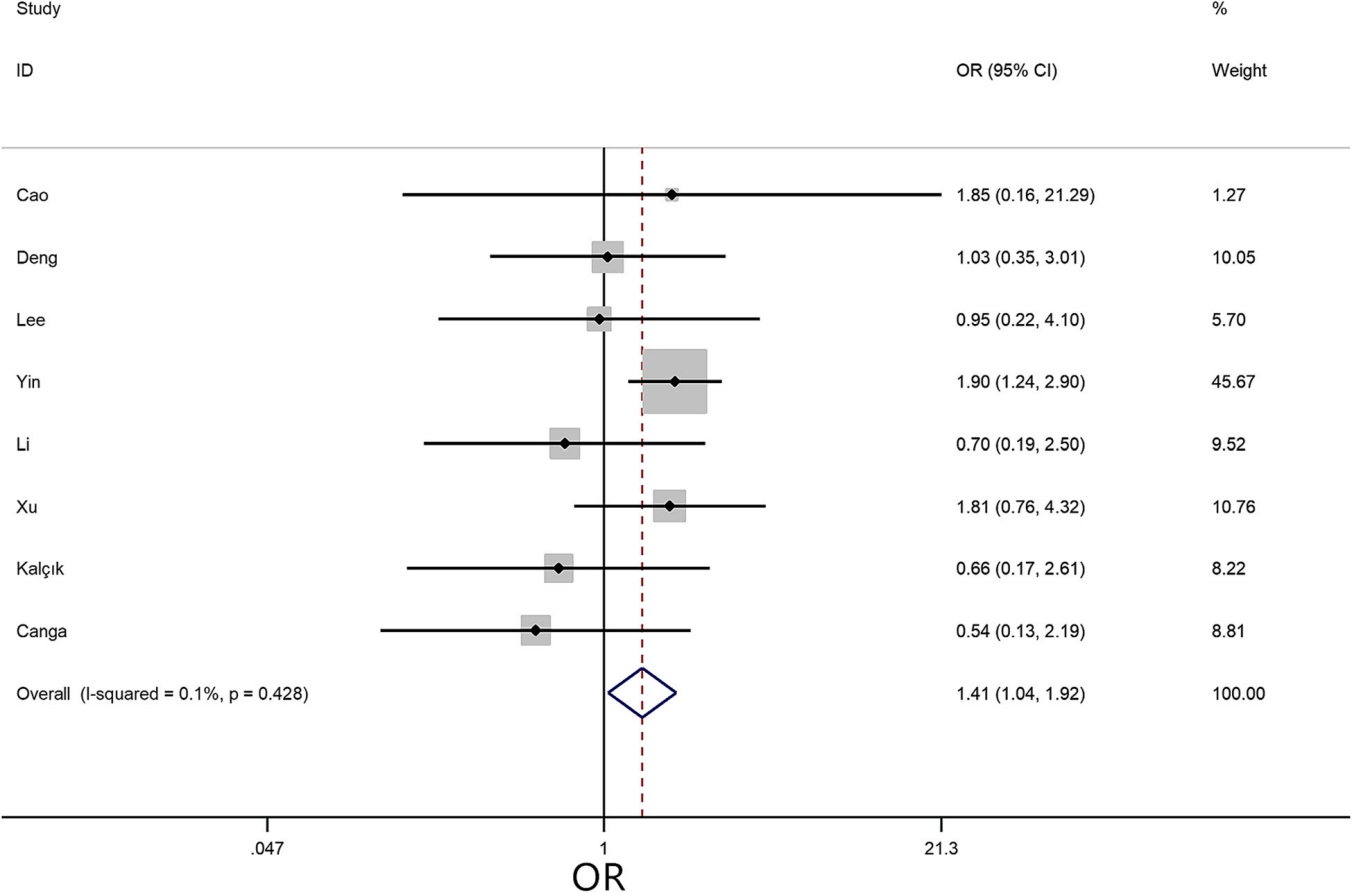
Forest plot for hypertension between embolism: 8 studies were included, I^2^ = 0.1%, fixed-effect model was adopted; the result showed hypertension a risk factor

A meta-analysis was performed to assess the 12 risk factors, of which NYHA class (I/II) (OR = 2.98, 95% CI = 1.66–5.33, *P* < 0.01) (Fig. 2) and hypertension (OR = 1.41, 95% CI = 1.04–1.92, *P* = 0.03) (Fig. 3) significantly increased the risk of embolism in CM patients. However, no statistically significant difference was detected in the meta-analysis with respect to sex (OR = 1.21, 95% CI = 0.96–1.53, *P* = 0.11), age (MD = -0.04, 95% CI = -3.64–3.56, *P* = 0.94), body mass index (BMI) (MD = 1.21, 95% CI = -0.43–2.84, *P* = 0.18), smoking (OR = 0.90, 95% CI = 0.64–1.28, *P* = 0.56), left ventricular ejection fraction (LVEF) (MD = 0.59, 95% CI = -0.20–1.38, *P* = 0.14), diabetes (OR = 1.32, 95% CI = 0.89–1.94, *P* = 0.16), hyperlipidemia (OR = 0.99, 95% CI = 0.53–1.85, *P* = 0.96), AF (OR = 1.25, 95% CI = 0.88–1.80, *P* = 0.22), valvular heart disease (OR = 0.76, 95% CI = 0.41–1.40, *P* = 0.38), and coronary heart disease (OR = 0.99, 95% CI = -0.87–2.85, *P* = 0.32).

### Tumor characteristics

**Fig. 4:**
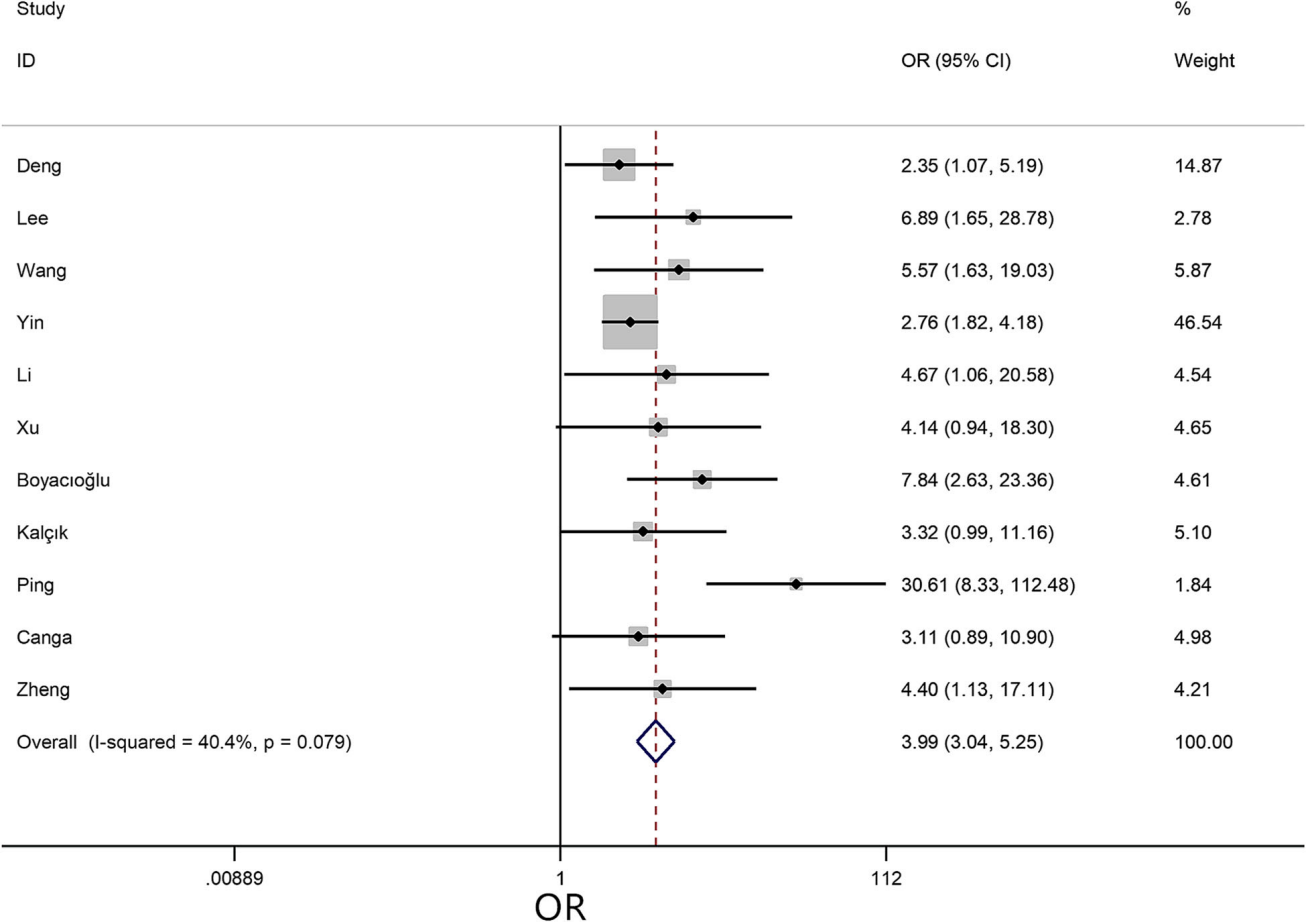
Forest plot for tumor surface between embolism: 11 studies were included, I^2^ = 40.4%, fixed-effect model was adopted; the result showed irregular surface is a risk factor

**Fig. 5:**
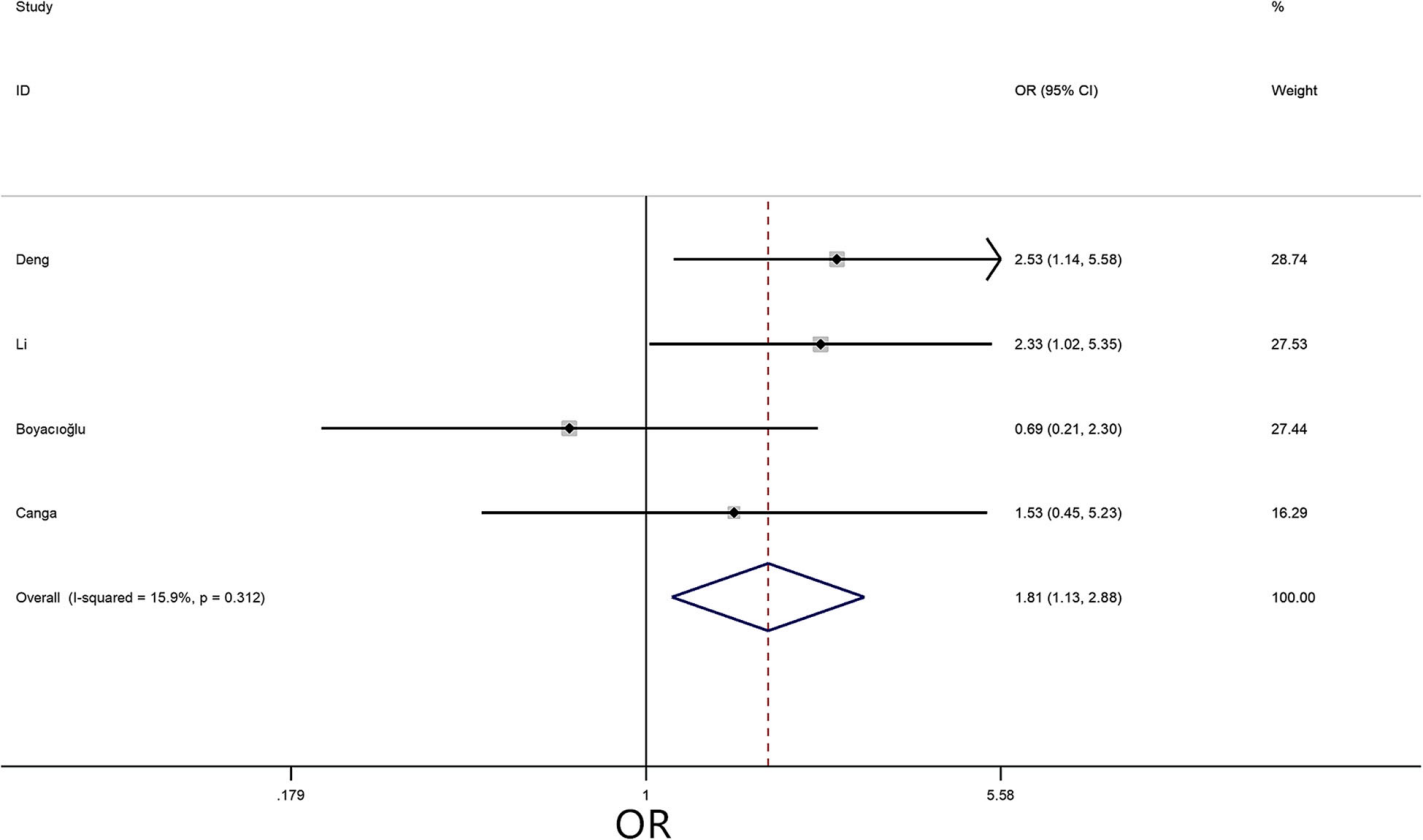
Forest plot for tumor location between embolism: 4 studies were included, I^2^ = 15.9%, fixed-effect model was adopted; the result showed atypical location is a risk factor

**Fig. 6:**
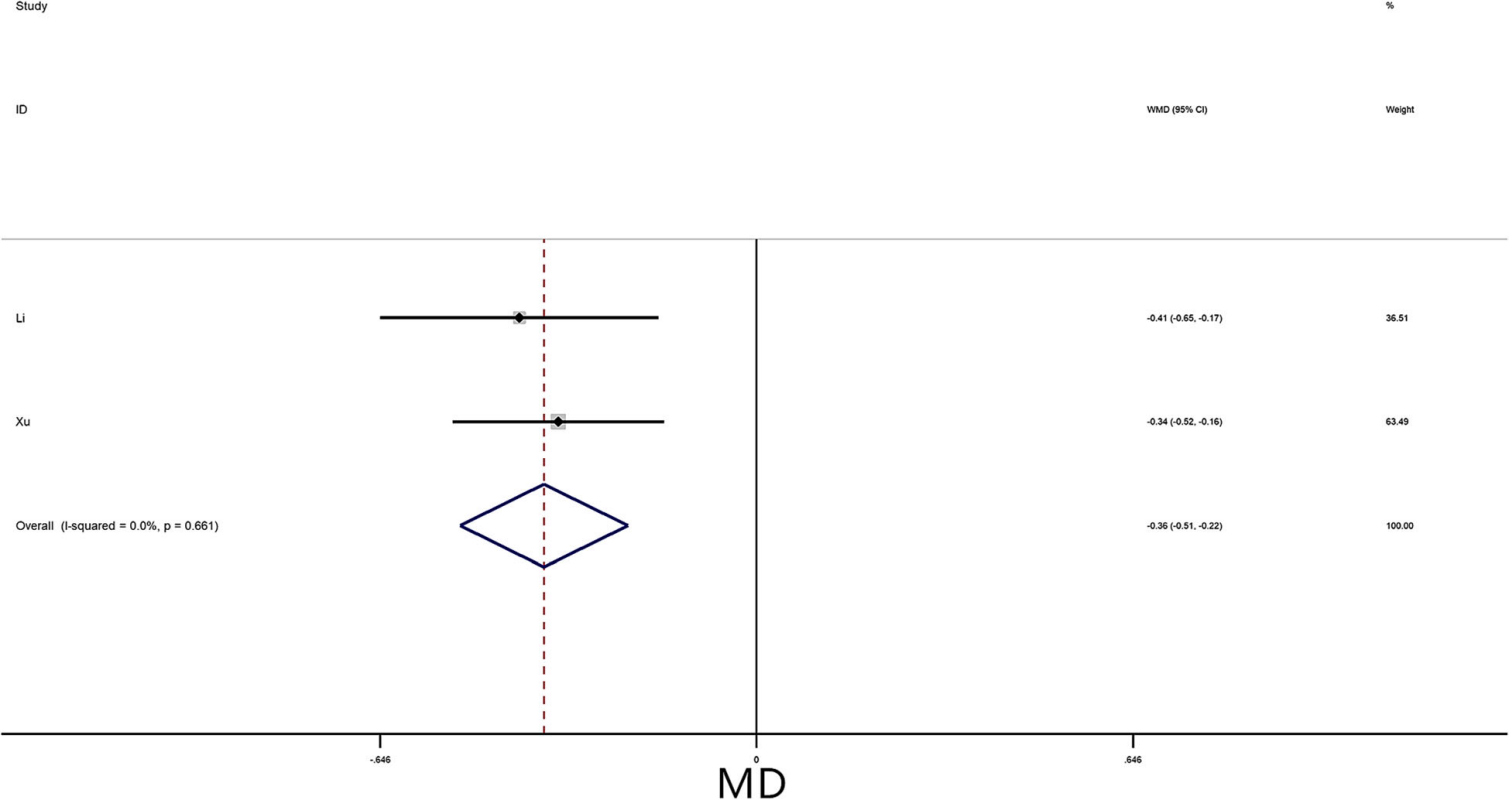
Forest plot for tumor base size between embolism: 2 studies were included, I^2^ = 0.0%, fixed-effect model was adopted; the result showed narrow base is a risk factor

A meta-analysis was performed for 4 risk factors. Of these, irregular tumor surface (OR = 3.99, 95% CI = 3.04–5.25, *P* < 0.01) (Fig. 4), atypical location (OR = 1.81, 95% CI = 1.13–2.88, *P* = 0.01) (Fig. 5), and narrow base (MD = -0.36, 95% CI = -0.51–-0.22, *P* < 0.01) (Fig. 6) significantly increased the risk of embolism in CM patients, while tumor size (MD = -0.10, 95% CI = -0.76–0.57, *P* = 0.78) was not associated with the condition.

### Hematological parameters

**Fig. 7:**
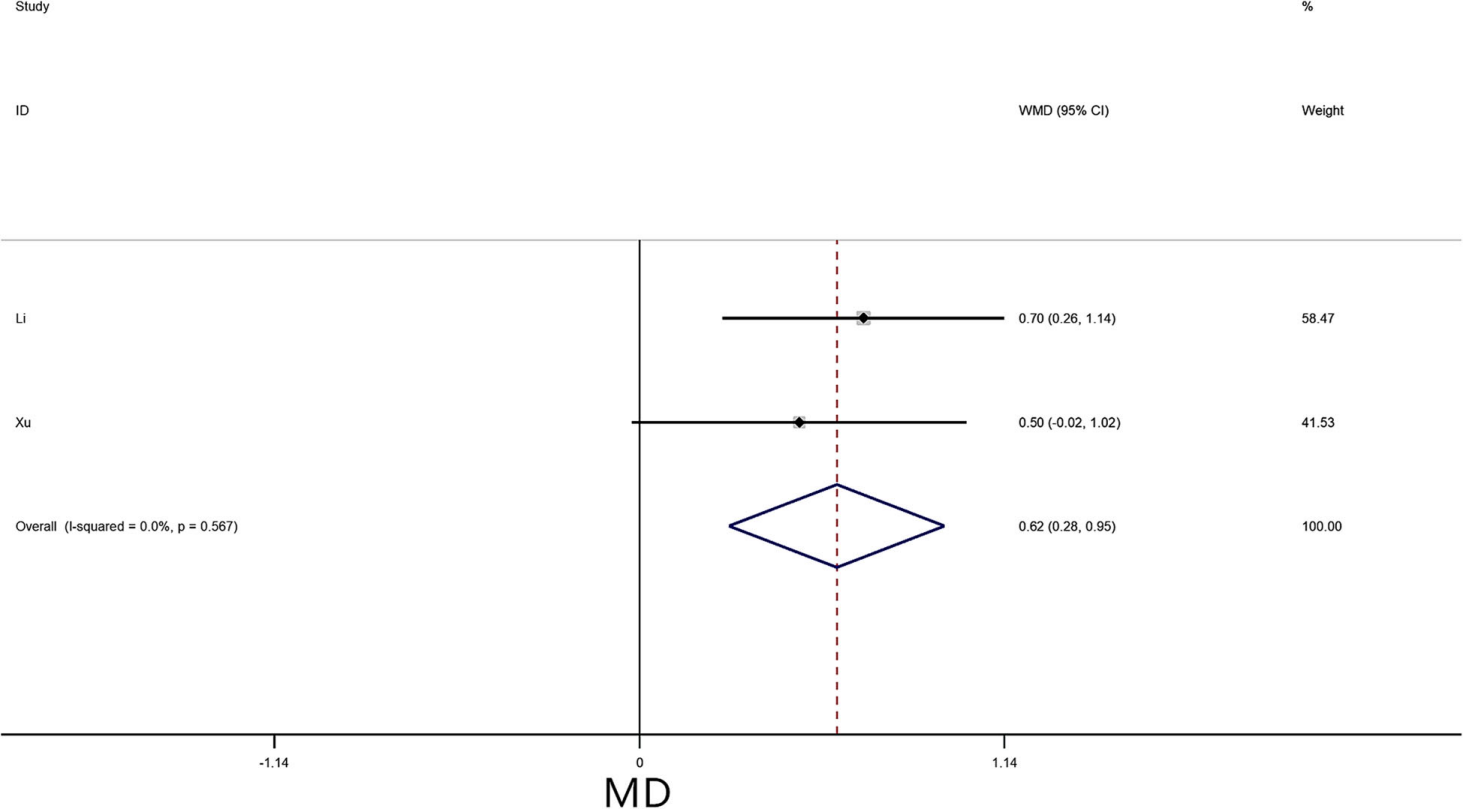
Forest plot for FIB between embolism: 2 studies were included, I^2^ = 0.0%, fixed-effect model was adopted; the result showed increased FIB is a risk factor

A meta-analysis was performed for 4 risk factors. Of these, increased fibrinogen (FIB) (MD = 0.62, 95% CI = 0.28–0.95, *P* < 0.01) (Fig. 7) significantly increased the risk of embolism in CM patients, while no statistically significant difference was detected in the meta-analysis with respect to PLT (MD = 9.95, 95% CI = -6.02–25.91, *P* = 0.22), WBC (MD = 0.18, 95% CI = -0.33–0.68, *P* = 0.49), and hemoglobin (HB) (MD = 1.65, 95% CI = -2.91–6.21, *P* = 0.48).

### Sensitivity analysis

High heterogeneity across studies was detected regarding age (I^2^ = 76.8%), BMI (I^2^ = 83.7%), and coronary heart disease (I^2^ = 84.2%). Sensitivity analysis showed a similar trend among various studies except for the study by Yin et al. [9]. After the exclusion of Yin’s study, the among-study heterogeneity was not detected (all I^2^ = 0.00%, all *P* > 0.05); also, the overall effect did not differ significantly (all *P* > 0.05).

In addition, tumor size had high heterogeneity (I^2^ = 83.3%), and sensitivity analysis showed a similar trend among studies except for the study by Kalçık et al. [13]. After this study was excluded, the among-study heterogeneity was found to be moderate (I^2^ = 58.6%, *P* = 0.03), while the overall effect did not differ significantly (*P* = 0.19).

### Publication bias

General considerations suggest that use of the Egger’s test with substantially fewer 10 studies would be unwise [22]; hence, the publication bias was assessed for sex and tumor surface. The Egger’s test not detect any publication bias on sex (*P* = 0.096) and tumor surface (*P* = 0.051).

## Discussion

In the current meta-analysis, a total of 20 potential factors were studied. The results suggested that the NYHA class, hypertension, tumor surface, tumor location, tumor base size, and FIB were independent risk factors associated with embolism.

Among patient characteristics, we found that 2/12 factors (NYHA class and hypertension) were significantly associated with embolism. The NYHA class is the most commonly used indicator of clinical response to cardiac function. The current study showed that better cardiac function (class I/II) was at a significantly higher risk of embolism than the worse function (class III/IV). This finding might be attributed to the fact that most embolic group patients were diagnosed after an acute embolic event; also, the tumor blockage symptoms were relatively less, and the overall cardiac function was better than that of non-embolic group. Conversely, the non-embolic group patients have a prolonged course of the disease and are likely to show hemodynamic changes; this phenomenon was associated with obstructive heart failure. However, the LVEF was not associated with embolism. We think this difference may attribute to the fact that some patients exist heart failure with preserved ejection fraction, and they also could reach NYHA class III/IV [23]. Hence, additional quantitative research about cardiac function is imperative. Some studies reported that hypertension might be a major risk factor of deep venous thromboembolism [24, 25]. Interestingly, the present study found that hypertension also was a risk factor of embolism in CM patients, which could be because high blood pressure increases the activity of PLT, making the blood hypercoagulable [26].

Regarding tumor characteristics, we found that three factors are related to embolism: tumor surface, location, and base. Macroscopically, the surface of the tumor is classified into two types [27, 28] as follows: Type 1 is characterized by an irregular surface and soft consistency, while type 2 presents a regular surface and compact consistency. Consistent with the previous studies [29–31], the current study found that embolic events are often associated with type 1 myxoma. This correlation might occur because the type 1 myxoma is prone to be friable, which leads to the shedding of tumor fragments into the bloodstream [13]. In addition, we found that atypical location is a risk factor of embolism. This conclusion is consistent with the result of the study by Deng et al. [6]. Thus, we hypothesized that the atypical location plays a significant role in hemodynamics than typical location. Lastly, the narrow tumor base was also a risk factor in this study owing to its great mobility, which tends to generate fragments [11].

In terms of hematological parameters, the present study designated increased FIB as a risk factor of embolism. It is a substrate for thrombin and is directly involved in the clotting process. In addition, FIB promotes the aggregation of PLT, increases blood viscosity, and aggravates red blood cell adhesion, all of which promote thrombosis [32]. Thus, we inferred that increased FIB promotes thrombosis on the surface of CM, which contributes to embolism.

To the best of our knowledge, this is the first meta-analysis to investigate the risk factors of embolism in patients with CM, with some practical clinical implications. Nevertheless, this meta-analysis also has some limitations. First, since all enrolled studies were retrospective case series, residual confounders and unidentified factors were inevitable in observational studies. Second, all the included studies originated from Asia, which might lead to selection bias. Third, the number of overlapped risk factors in each of the studies was small for a comprehensive statistical analysis. Finally, with respect to the tumor size, the data of our meta-analysis showed high heterogeneity. Although one major source of heterogeneity was detected by sensitivity analysis, other differences between the studies should be considered.

## Conclusions

This systematic review and meta-analysis identified the following significant risk factors of embolism for CM patients: NYHA class I/II, hypertension, irregular tumor surface, atypical tumor location, the narrow base of tumor, and increased FIB. For CM patients with these factors, early surgery may be beneficial for preventing embolism.

## Supplementary information

**Additional file 1.**

**Additional file 2.**

**Additional file 3.**

## Data Availability

The datasets used and/or analyzed during the current study are available from the corresponding author on reasonable request.

## Abbreviations

CM: Cardiac myxoma
OR: Odds ratio
MD: Mean difference
CI: Confidence interval
NYHA: New York heart association
FIB: Fibrinogen
BMI: Body mass index
LVEF: Left ventricular ejection fraction
AF: Atrial fibrillation
PLT: Platelet count
WBC: White blood cells
HB: Hemoglobin
