# Supplementary material for: Risk factors of embolism for the cardiac myxoma patients: a systematic review and metanalysis
Source: BMC Cardiovasc Disord. 2020 Jul 25;20:348. doi: 10.1186/s12872-020-01631-w (PMC7382866; doi:10.1186/s12872-020-01631-w)
Supplement: Supplementary file 1 — Additional file 1. [file 12872_2020_1631_MOESM1_ESM.docx]

Additional file 1. Search string

The search strings in different databases are showed as follows.

**(1) PubMed**

#1: "Myxoma"[Mesh]

#2: myxomas [Title/Abstract] OR angiomyxoma [Title/Abstract] OR angiomyxomas [Title/

Abstract]

#3: #1 OR #2

#4: "Embolism"[Mesh]

#5: embolisms [Title/Abstract]) OR embolus [Title/Abstract]

#6: #4 OR #5

#7: "Heart"[Mesh]

#8: hearts [Title/Abstract]

#9: #7 OR #8

#10: #3 AND #6 AND #9

**(2) Embase**

#1: ‘myxoma’/exp

#2: ‘myxomas’:ab,ti OR ‘angiomyxoma’:ab,ti OR ‘angiomyxomas’:ab,ti

#3: #1 OR #2

#4: ‘embolism’/exp

#5: ‘embolisms’:ab,ti OR ‘embolus’:ab,ti

#6: #4 OR #5

#7: ‘heart’/exp

#8: ‘hearts’:ab,ti

#9: #7 OR #8

#10: #3 AND #6 AND #9

**(3) Web of science**

TS=("heart") AND TS=("myxoma") AND TS=("embolism")

**(4) Cochrane Library database:**

#1:(myxoma): ti,ab,kw OR (myxomas):ti,ab,kw OR (angiomyxoma):ti,ab,kw OR (angiomyxomas):ti,ab,kw

#2:(embolism): ti,ab,kw OR (embolisms):ti,ab,kw OR (embolus):ti,ab,kw

#3:(heart): ti,ab,kw OR (hearts):ti,ab,kw

#4: #1 AND #2 AND #3

**(5) CNKI**

( (主题=粘液瘤 或者 题名=粘液瘤 或者 v_subject=中英文扩展(粘液瘤,中英文对照) 或者 title=中英文扩展(粘液瘤,中英文对照)) 或者 (主题=黏液瘤 或者 题名=黏液瘤 或者 v_subject=中英文扩展(黏液瘤,中英文对照) 或者 title=中英文扩展(黏液瘤,中英文对照)) ) 并且 (主题=心脏 或者 题名=心脏 或者 v_subject=中英文扩展(心脏,中英文对照) 或者 title=中英文扩展(心脏,中英文对照)) 并且 (主题=栓塞 或者 题名=栓塞 或者 v_subject=中英文扩展(栓塞,中英文对照) 或者 title=中英文扩展(栓塞,中英文对照)) (模糊匹配)

**(6) Wan Fang**

（主题：（粘液瘤+黏液瘤）*主题：（心脏）*主题：（栓塞））

**(7) VIP**

((题名或关键词=心脏粘液瘤 OR 题名或关键词=心脏黏液瘤) AND 题名或关键词=栓塞)
