# Supplementary material for: Risk factors of embolism for the cardiac myxoma patients: a systematic review and metanalysis
Source: BMC Cardiovasc Disord. 2020 Jul 25;20:348. doi: 10.1186/s12872-020-01631-w (PMC7382866; doi:10.1186/s12872-020-01631-w)
Supplement: Supplementary file 2 — Additional file 2. [file 12872_2020_1631_MOESM2_ESM.pdf]

MedSci

Editing by professional editors

CERTIFICATE OF ENGLISH EDITING

Date:1st July 2020

This document certifies that the manuscript titled "Risk factors of embolism for the cardiac myxoma patients: A systematic review AND metanalysis" was edited for proper English language, grammar, punctuation, spelling, and overall style by one or more of the highly qualified native English speaking editors at MedSci. Neither the research content nor the authors' intentions were altered in any way during the editing process.

Documents receiving this certification should be English-ready for publication - however, the author has the ability to accept or reject our suggestions and changes. To verify the final MedSci edited version, please visit our verification page. If you have any questions or concerns over this edited document, please contact MedSci at editing@medsci.cn

**Manuscript title:**	Risk factors of embolism for the cardiac myxoma patients: A systematic review AND metanalysis
**First Author:**	Yanna Liu
**Code:**	0107-42DA-790D-E1BD-492E

This certificate may be verified at www.medsciediting.com/certificate/

MedSci affiliated to BIOON Group, offers the services in paper evaluation, professional translation, language editing and publication support.

MedSci comprises a network of more than 300 editors with a biomedical background. All our editors must meet strict selection criteria. We require that each editor has completed or is attending a PhD graduate program at one of the top universities in the world. He or she must have proven written communication skills and undergo a comprehensive training period under the instruction of a managing editor. All editors are native English speakers.

We provide high quality services at the comparable price in the industry. For more information, please visit www.medsciediting.com or contact us by email at editing@medsci.cn
